# Chitinase-3 like-protein-1 function and its role in diseases

**DOI:** 10.1038/s41392-020-00303-7

**Published:** 2020-09-14

**Authors:** Ting Zhao, Zhongping Su, Yingchang Li, Xiaoren Zhang, Qiang You

**Affiliations:** 1 Affiliated Cancer Hospital & Institute, Guangzhou Medical University, Guangzhou, China; 2 Department of Biotherapy, Department of Geriatrics, Second Affiliated Hospital of Nanjing Medical University, Nanjing, China; 3 Key Laboratory of Cell Homeostasis and Cancer Research of Guangdong Higher Education Institutes, Guangzhou Medical University, Guangzhou, China

**Keywords:** Pathogenesis, Molecular biology

## Abstract

Non-enzymatic chitinase-3 like-protein-1 (CHI3L1) belongs to glycoside hydrolase family 18. It binds to chitin, heparin, and hyaluronic acid, and is regulated by extracellular matrix changes, cytokines, growth factors, drugs, and stress. CHI3L1 is synthesized and secreted by a multitude of cells including macrophages, neutrophils, synoviocytes, chondrocytes, fibroblast-like cells, smooth muscle cells, and tumor cells. It plays a major role in tissue injury, inflammation, tissue repair, and remodeling responses. CHI3L1 has been strongly associated with diseases including asthma, arthritis, sepsis, diabetes, liver fibrosis, and coronary artery disease. Moreover, following its initial identification in the culture supernatant of the MG63 osteosarcoma cell line, CHI3L1 has been shown to be overexpressed in a wealth of both human cancers and animal tumor models. To date, interleukin-13 receptor subunit alpha-2, transmembrane protein 219, galectin-3, chemo-attractant receptor-homologous 2, and CD44 have been identified as CHI3L1 receptors. CHI3L1 signaling plays a critical role in cancer cell growth, proliferation, invasion, metastasis, angiogenesis, activation of tumor-associated macrophages, and Th2 polarization of CD4^+^ T cells. Interestingly, CHI3L1-based targeted therapy has been increasingly applied to the treatment of tumors including glioma and colon cancer as well as rheumatoid arthritis. This review summarizes the potential roles and mechanisms of CHI3L1 in oncogenesis and disease pathogenesis, then posits investigational strategies for targeted therapies.

## Introduction

Glycoside hydrolase family 18 includes chitinases and non-enzymatic chitinase-like proteins (CLPs), both of which bind chitin, a polysaccharide chain composed of N-acetylglucosamine repeats and present in arthropods and other taxa as a major structural polymer. While chitinases cleave chitin, CLPs do not possess this enzymatic activity. chitinase-3 like-protein-1 (CHI3L1), one of the CLPs, also has been named YKL-40 in humans and breast regression protein 39 (BRP-39) in mice, is common in both prokaryotes and eukaryotes. Following its initial discovery in the culture supernatant of the osteosarcoma cell line MG63,^1^ it was subsequently detected in human chondrocytes, synoviocytes, and vascular smooth muscle cells.^2,3^ In fact, CHI3L1 is produced by a multitude of cells, including macrophages, neutrophils, fibroblast-like cells, hepatic stellate cells, endothelial cells, and cancer cells.^4–9^ For the moment, extracellular matrix (ECM) changes, miRNAs, growth factors, cytokines, stress, and drugs have been reported to be effective regulators of the synthesis and secretion of CHI3L1.^10–13^

The crystal structure of the native protein shows that CHI3L1 consists of a (β/α)_8_-barrel fold with a β+α domain insertion,^14,15^ which are essential for its functions in both physiological and pathological processes. CHI3L1 plays a crucial role in protecting against pathogens, antigen-induced and oxidant-induced injury responses, inflammation, and tissue repair and remodeling by regulating a variety of essential biological processes including oxidant injury, apoptosis, pyroptosis, inflammasome activation, Th1/Th2 inflammatory balance, M2 macrophage differentiation, dendritic cell (DC) accumulation, TGF-β1 expression, ECM regulation, and parenchymal scarring.^16–19^

CHI3L1 is overexpressed in many human cancer types and animal tumor models, for instance, oligodendroglia, glioblastoma, osteosarcoma, sarcoma, colon, and gastric cancers (GCs).^20–27^ Elevated serum levels of CHI3L1 have been found to be associated with poor prognosis and shorter survival in patients with metastatic cancer.^28^ Consequently, CHI3L1 has been increasingly proposed as a sensitive biomarker and an attractive therapeutic target for several certain types of cancers.^29^ Interleukin-13 receptor subunit alpha-2 (IL-13Rα2), and its interactions with transmembrane protein 219 (TMEM219), galectin-3 (Gal-3), and CD44 have been identified to be receptors of CHI3L1 respectively.^30–32^ Additionally, collaboration of integrin α_v_β_3_ with syndecan-1 (Syn-1), and coordination between integrin α_v_β_5_ and Syn-1 are newly discovered interactions that are activated by CHI3L1 to trigger signaling pathways and downstream cascades.^33,34^ However, the underlying mechanisms involved are unclear, with key downstream targets remaining to be deeply identified.

In this review, we discuss the sources, regulation, crystal structure, biological functions, and the potential roles of CHI3L1 in oncogenesis and disease pathogenesis. Accordingly, we have also illustrated various applied targeting therapies and proposed certain theoretically feasible strategies.

## Genetic variants and crystal structure of CHI3L1

The human *CHI3L1* gene is located on chromosome 1q31–1q32, comprising 7498 base pairs and 10 exons. It spans ~8 kbp of genomic DNA.^35^ Three promoter single-nucleotide polymorphisms (SNPs) (rs4950928, rs10399805, and rs10399931), 1 non-synonymous SNP (rs880633), and four intronic SNPs (rs1538372, rs2071579, rs946259, and rs2275353) in *CHI3L1* have been found to be linked with its serum level in the general population, at or below genome-wide association significance levels.^36^ Accordingly, genetic variation in *CHI3L1* is closely associated with the incidence and prognosis of multiple inflammatory and neoplastic diseases.^37,38^ In asthma patients with European ancestry, the risk allele A at rs12141494 has been found to be associated with higher levels of CHI3L1 in the airway and severe asthma.^39^ Similarly, in the southwest Chinese Han population, the rs10399931 CT/TT genotypes of CHI3L1 were associated with increased risk of asthma.^40^

**Fig. 1:**
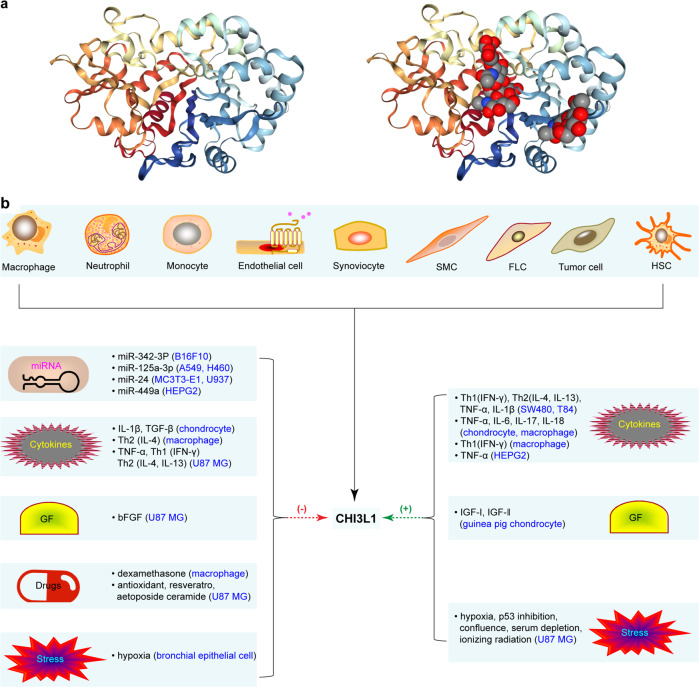
Crystal structure, source, and expression regulation of CHI3L1. **a** A long carbohydrate-binding cleft is present at the C-terminal side of the beta-strands. Binding of chitotetraose is shown in the groove. **b** CHI3L1 is synthesized and secreted by a multitude of cells. CHI3L1 expression is regulated by miRNAs, cytokines, growth factors (GF), drugs, and stress. SMC smooth muscle cell, FLC fibroblast like cells, HSC hepatic stellate cell

CHI3L1 is an indispensable member of the glycoside hydrolase family 18,^41^ which binds to chitin, but lacks enzymatic activity.^41,42^ Crystal diffraction studies revealed that the three-dimensional structure of CHI3L1 consisted of an (β/α)_8_-barrel domain with a second domain composed of six antiparallel β-strands, with one α-helix (α + β) domain inserted after strand β7 (Fig. 1a: 10.2210/pdb1NWU/pdb).^14,15^ Additionally, a 43-residue carbohydrate-binding cleft was found exposed at the C-terminal side of the β-strands in the (β/α)_8_ barrel.^43^ Essentially, the protein–carbohydrate interactions are dominated by stacking of the sides.^43^ This structure suggests that CHI3L1 acts as a sensor to turn on innate defenses and regulates inflammatory responses as a consequence of infection, which can also contribute to tumorigenesis.

## Source and expression of CHI3L1

CHI3L1 was originally discovered in the culture supernatant of MG63 cell lines.^1^ It was also identified in the monolayer or explant culture of human articular chondrocytes, and was termed human cartilage glycoprotein-39 (HC-gp39).^2^ It has been named YKL-40, owing to the three N-terminal amino-acid residues present in the secreted form: tyrosine (Y), lysine (K), and leucine (L) with a molecular weight of 40 kDa.^44^

CHI3L1 is produced by a multitude of cells including macrophages, neutrophils, stem cells, bone cells, synoviocytes, chondrocytes, fibroblast-like cells, endothelial cells, vascular smooth muscle cells, hepatic stellate cells, mammary epithelial cells, and cancer cells.^42,45–48^ Overexpression of CHI3L1 has been observed in a number of inflammatory conditions including asthma, sepsis, diabetes, cirrhosis, preeclampsia, rheumatoid arthritis, and coronary artery disease.^33,38,49–54^

## Regulation of CHI3L1 expression

At the gene level, miR-24 shows strong complementarity and a high degree of species conservation with respect to its binding sites within the 3′ UTR of the CHI3L1 mRNA.^55^ Consequently, miR-24 was found to downregulate the expression of CHI3L1 in *S. aureus*-infected MC3T3-E1 cells^56^ and block the induction of CHI3L1, which attenuates aortic vascular inflammation and the development of murine abdominal aneurysms.^57^ Similarly, following hepatitis C virus (HCV) infection miRNA-449a was found to modulate the expression of CHI3L1 by targeting the components of the NOTCH signaling pathway.^58^ Recent studies have revealed that miR-342-3p suppresses NF-κB-mediated CHI3L1 expression during vascular inflammation and atherosclerosis to prevent memory dysfunction.^59^

At the cellular level, cytokines, growth factors, cellular and ECM factors, drugs, and stress are effective regulators of CHI3L1 production. For example, changes in the ECM of chondrocytes has been linked to the synthesis and secretion of CHI3L1 [54]. IL-1β and TGF-β have been reported to inhibit the expression of *CHI3L1* mRNA in human chondrocytes and cartilage explant cultures.^11^ Conversely, IL-1β facilitates the production of CHI3L1 in SW480 and T84 cell lines. A similar effect has been reported to be caused by TNFα, Th1 (IFN-γ), and Th2 (IL-4 and IL-13).^60^ CHI3L1 production has also been reported to be enhanced by the key Th1 cytokine, IFN-γ, and suppressed by the Th2 cytokine, IL-4, in activated macrophages.^13^ Stimulation by IL-6, IL-17, and IL-18 has resulted in CHI3L1 secretion from freshly isolated chondrocytes.^5,6,61^ However, IL-7, IL-11, IL-12, PDGF, and bFGF have no such effect on CHI3L1 synthesis and secretion in human chondrocyte and cartilage explant cultures.^11^ IGF-I and IGF-II stimulate CHI3L1 synthesis in guinea pig chondrocytes, but have no effect on human chondrocytes.^2,12^ CHI3L1 is overexpressed in U87 MG cells cultured under stresses including hypoxia, ionizing radiation, confluence, etoposide ceramide, p53 inhibition, antioxidant treatment, and serum depletion. Conversely, TNFα and bFGF exert an inhibitory influence on CHI3L1 production^6^ (Table 1, Fig. 1b).

**Table 1:** Regulation of CHI3L1 expression

Types		Targeted cell		References
Gene level	miR-342-3p	B16F10 cell lines	↓	^263^
	miR-125a-3p	A549, H460 cell lines	↓	^264^
	miR-24	MC3T3-E1 cell, U937 cell	↓	^55,56,149^
	miR-449a	HEPG2 cells	↓	^58^
Cellular level	ECM		↑	^265^
Cytokines	Th1 (IFN-γ), Th2 (IL-4, IL-13), TNFα, IL-1β	SW480 and T84 cell lines	↑	^29,60,118^
	IL-6, IL-17, IL-18, TNFα	Chondrocytes, macrophages	↑	^266–268^
	IL-1β, TGF-β	Chondrocytes	↓	^11^
	Th1 (IFN-γ)	Macrophages	↑	^13^
	Th2 (IL-4)	Macrophages	↓	^13^
	TNFα	HEPG2 cells,	↑	^58^
		Human skeletal muscle cells	↑	^9^
	TNFα, Th1 (IFN-γ),	U87 MG cell lines	↓	^6^
	Th2 (IL-4, IL-13)			
Growth factors	IGF-I, IGF-II	Guinea pig chondrocytes	↑	^2,12^
	bFGF	U87 MG cell lines	↓	^6^
Drugs	Dexamethasone,	Macrophages	↓	^13^
	Etoposide ceramide,	U87 MG cell lines	↓	^6,258^
	Antioxidant, resveratrol			
Stress	Hypoxia, confluence, p53 inhibition, serum depletion, lonizing radiation	U87 MG cell lines	↑	^6^
	Hypoxia	Bronchial epithelial cells	↓	^269^

## Biological activities of CHI3L1

### CHI3L1 stimulates cell growth and proliferation

CHI3L1 promotes the growth and proliferation of guinea pig chondrocytes, rabbit chondrocytes, and synovial cells.^61^ In humans, CHI3L1 facilitates the growth of synovial cells, articular chondrocytes, skin, and fetal lung fibroblasts through the phosphorylation of MAPK and Akt signaling.^5^ Accordingly, CHI3L1 silencing decreases the proliferation of HEK293 and U87 cells.^41,62,63^ During asthma, CHI3L1 increases bronchial smooth-muscle cell growth and proliferation through PAR-2-dependent, Akt-dependent, Erk-dependent, and p38-dependent mechanisms.^33^ In the tissue repair and remodeling of asthma, CHI3L1 was found to induce IL-8 expression in bronchial epithelium, via MAPK (JNK and Erk) and NF-κB pathways, to stimulate bronchial smooth muscle cell proliferation.^64^ CHI3L1 also works synergistically with IGF-1 to stimulate the growth of fibroblasts by initiating MAPK/Erk1/2 and phosphatidylinositol 3 kinase (PI3K) signaling cascades that play a major role in tissue fibrosis.^5^ Essentially, both MAPK and PI3K pathways are important in mitogenesis, growth, proliferation, apoptosis, and cancer-cell transformation.^65^

CHI3L1 has a chemotactic effect on vascular endothelium and smooth-muscle cells during tissue injury and remodeling, inflammation, and fibrosis.^6^ It regulates the morphology of vascular endothelial cells by stimulating endothelium tubulogenesis and vascular smooth muscle cell migration and adhesion.^66^

### CHI3L1 promotes cell survival

CHI3L1 has been found to protect cardiomyocytes from apoptosis during ischemia-reperfusion injury.^67^ It also has a pro-inflammatory function in reducing inflammatory cell apoptosis and death, by inhibiting Fas expression through the phosphorylation of protein kinase B (PKB)/Akt.^68^ Similarly, CHI3L1 has been reported as a potent inhibitor of death receptor-induced inflammatory cell apoptosis, which is accomplished through Fas expression inhibition, PKB/Akt activation, and Faim 3 induction.^69^ One group has reported that phosphorylation of PKB/Akt correlates closely with cell apoptosis and survival.^70^

CHI3L1 inhibition by shRNA increases cell death triggered by several anticancer drugs, including cisplatin, etoposide, and doxorubicin, whereas overexpressed CHI3L1 exhibits the opposite effect in glioblastoma U87 MG cells.^41^ Particularly in late-stage glioblastoma, CHI3L1 regulates tumorigenesis by interrupting the pathways leading to apoptosis.^6^ Additionally, CHI3L1 also protects cancer cells from apoptosis by remodeling the ECM to create a good substrate for tumor growth and progress.^71^

### CHI3L1 drives immune cell activation and differentiation

CHI3L1 has a significant impact on macrophage differentiation, DC accumulation, and Th1/Th2 balance.^18^ In contrast to other monocyte/macrophage markers, CHI3L1 is not found in monocytes, but is strongly induced during the late stages of differentiation in human macrophages, where nuclear Sp1 binds to the CHI3L1 promoter to facilitate the late stages of human macrophage maturation.^72^

CHI3L1 plays a significant role in the pathogenesis of CD4 T^+^ cell polarization and Th2 inflammation. CHI3L1-deficient CD4^+^ T cells differentiate into Th1 cells.^73^ Essentially, CHI3L1 is expressed in activated T cells and Th2 cells, regulating Th1 and Th2 differentiation through IFN-γ signaling via the IFN-γ–STAT1 axis.^73^ Therefore, CHI3L1 is a regulator of Th1 polarization and cytotoxic T lymphocyte (CTL) expression, which serve as potential therapeutic targets to enhance anti-tumor immunity. CHI3L1 expression is greatly enhanced during Th2 inflammatory responses induced by ovalbumin, aluminum hydroxide, house dust, and mites, while *CHI3L1* knockout mice exhibit reduced Th2 inflammation.^69^ CHI3L1 expression is induced by a high-fat diet and contributes to the genesis of obesity and asthma by the inhibition of sirt1 expression.^74^ CHI3L1 is expressed in a time-dependent manner during the differentiation and maturation of monocyte-derived DCs, and distributed in the cytoplasm and nucleus of both immature and mature DCs.^75^

### CHI3L1 regulates the synthesis and degradation of the ECM

The ECM, a collection of extracellular molecules, provides structural and biochemical support to surrounding cells,^76^ also participating in gene expression, cellular differentiation, cell adhesion, and intercellular communication.^77,78^ Increased ECM degradation promotes cell migration, invasion, and tumorigenesis. The ECM barrier represents the first obstacle for invasive tumor migration and establishment of metastases.^79^ It has been shown that CHI3L1 inhibits the degradation of type I collagen and hyaluronic acid. It also affects the enzymatic activity of matrix metalloproteinases (MMPs), thereby influencing the extent of cell adhesion and migration,^80^ influencing tissue remodeling, fibrosis, and tumorigenesis. CHI3L1 suppresses the expression of E-cadherin while enhancing the activity of MMP-9 and cell motility, to mediate mammary tissue remodeling during involution.^81^ Essentially, the absence of E-cadherin function, which leads to decreased cell–cell adhesion, transmits signals which actively promote tumor-cell invasion and metastasis.^82^ Likewise, activated MMP-9 enhances the invasion of the cultured cells by degrading ECM.^83^ Moreover, CHI3L1 inhibits IL-1-induced and TNF-α-induced secretion of MMPs (MMP-1, MMP-3, and MMP-13), which depends on the reduced activation of the p38 and SAPK/JNK pathways.^84^ The expression of MMP-1, MMP-3, and MMP-13 has been primarily detected in cartilage, where CHI3L1 targets and degrades proteoglycan, collagen, osteonectin, and perlecan to facilitate the progression of osteoarthritis (OA).^85^ The expression and enzymatic activity of MMP-2 has been reported to be markedly reduced in CHI3L1-silenced glioma cells.^41^ MMP-2 localization on the surface of invasive cells facilitates cell invasion by regulating matrix degradation and motility.^86^ Overall, CHI3L1 plays a crucial role in ECM regulation, which has a large impact on tissue remodeling and invasive cancer progression.

## Specific receptors, ligands, and interacting complexes of CHI3L1

### IL-13Rα2

IL-13Rα2, also known as CD213A2 and cancer/testis antigen 19 (CT19), is a glycosylated transmembrane protein which is highly expressed in several cancer types including glioblastoma,^87^ breast cancer,^88^ ovarian carcinoma,^89^ and colorectal cancer.^90^ It has been successfully applied as a therapeutic target of chimeric antigen receptor (CAR)-engineered T cells in a patient with recurrent multifocal glioblastoma.^91^ IL-13Rα2 has a short cytoplasmic motif that lacks the conserved box 1 region necessary for signal transduction. IL-13 binds IL-13Rα2 with high affinity and acts as a decoy receptor to inhibit response to IL-13 through the IL-13Rα1/IL-4Rα heterodimer.^92,93^

**Fig. 2:**
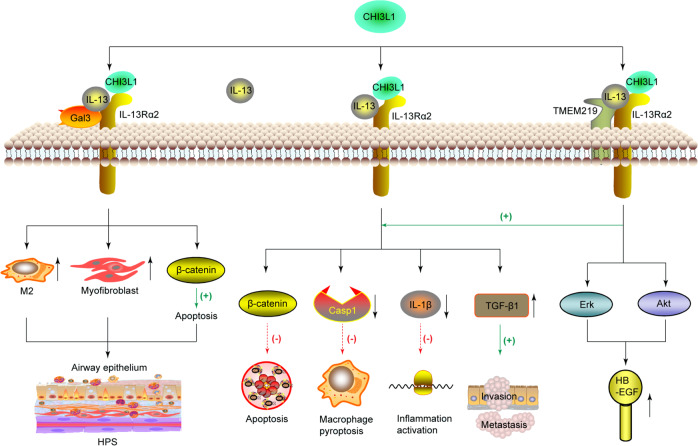
CHI3L1 interacts with IL-13-IL-13Rα2 to form a multimeric complex, and synergistically interacts with TMEM219 and Gal-3, respectively. CHI3L1 binds IL-13Rα2 to activate the Erk, Akt, and Wnt/β-catenin pathways to regulate apoptosis, pyroptosis, inflammasome activation, antibacterial responses, and malignancy metastasis. The binding ability of IL-13 with IL-13Rα2 is increased in the presence of TMEM219, thereby enhancing the anti-apoptosis response induced by CHI3L1 stimulation. Gal-3 interacts with CHI3L1–IL-13-IL-13Rα2 complex to compete with TMEM219 for IL-13Rα2 binding to diminish the anti-apoptotic role of CHI3L1. HPS Hermansky–Pudlak syndrome, HB-EGF heparin-binding EGF-like growth factor

CHI3L1 forms a multimeric complex with IL-13Rα2 and IL-13 to activate the MAPK/Erk, Akt, and Wnt/β-catenin cell signaling pathways to regulate apoptosis, oxidant injury-induced cell death, *Streptococcus pneumonia*-induced macrophage pyroptosis and inflammasome activation, antibacterial response, and melanoma metastasis.^18^ A recent study reveals that N-glycosylation is a critical determinant of CHI3L1 and IL-13 binding to IL-13Rα2.^94^ CHI3L1 and IL-13 do not compete for IL-13Rα2 binding and signaling, and they do not bind to identical locations on IL-13Rα2. The elimination of IL-13Rα2 partially abrogates specific CHI3L1 effector responses, suggesting the presence of other receptors^18^ (Fig. 2).

### TMEM219

TMEM219, also known as insulin-like growth factor-binding protein 3 receptor (IGFBP-3R), is a protein that acts as a cell death receptor for IGFBP-3 in breast and prostate cancers.^95^ It has been identified as a binding partner for IL-13Rα2 in the formation of the CHI3L1-IL-13Rα2–TMEM219 complex.^30^ The affinity of IL-13 with IL-13Rα2 has been reported to increase in the presence of TMEM219, which does not bind to IL-13Rα2. On CHI3L1 stimulation, TMEM219 enhances the expression of heparin-binding EGF-like growth factor (HB-EGF) on epithelial cells and macrophages through the activation of the MAPK/Erk and PKB/Akt pathways^30^ (Fig. 2).

### Galectin-3

The β-galactoside-binding protein Gal-3 is expressed at high levels in the nucleus, cytoplasm, and extracellular milieu of cells including lung epithelial cells.^96^ Gal-3 physically interacts with IL-13Rα2 and CHI3L1 to compete with TMEM219 for IL-13Rα2 binding and diminish the anti-apoptotic role of CHI3L1.^31^ Upon accumulation in the extracellular space, Gal-3 drives the apoptosis of primary lung epithelial cells. Conversely, the intracellular expression of Gal-3 has a dominating influence on M2 macrophage differentiation and myofibroblast proliferation, thereby contributing to exaggerated injury and fibroproliferative repair responses in Hermansky–Pudlak syndrome (HPS)^31^ (Fig. 2).

### Chemoattractant receptor-homologous molecule expressed on Th2 cells (CRTH2)

CHI3L1 interacts with the prostaglandin D2 receptor CRTH2 to enhance collagen accumulation in HPS1 mutant cells and promote the lung tissue fibrotic response. In HPS patients, the membrane expression of IL-13Rα2 is decreased.^97^ In normal individuals, however, CHI3L1 ameliorates epithelial cell apoptosis and lung injury in an IL-13Rα2-dependent manner.

### CD44

**Fig. 3:**
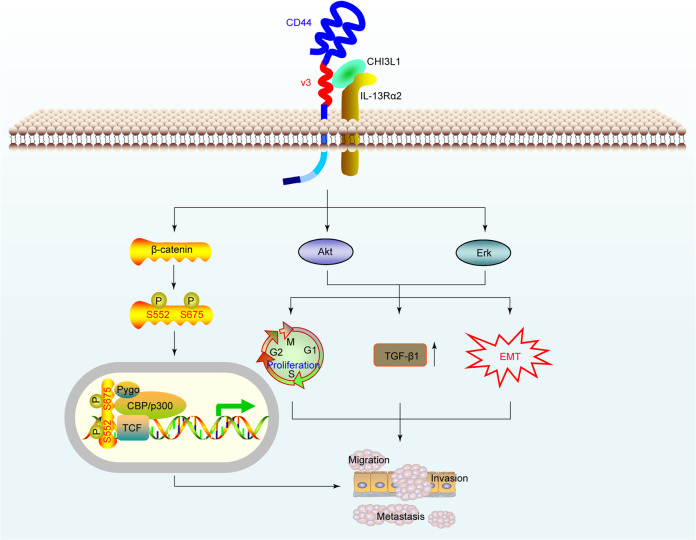
CHI3L1 physically interacts with the CD44 to promote GC invasion and metastasis. CHI3L1 is highly expressed in GC tissues and patient sera, and interacts with CD44v3 to activate the Akt, Erk, and β-catenin signaling pathways, contributing to GC progression

CD44, a cell-surface transmembrane glycoprotein, has been recognized as a key signaling regulator of cell growth, survival, and differentiation.^98^ Recently, our group reported that CHI3L1 physically interacts with CD44 to activate the Erk and Akt pathways, along with phosphorylation of β-catenin at Ser552 and Ser675.^32^ Interestingly, CD44v3 peptide and protein, but neither CD44v6 peptide nor CD44s protein, bound to CHI3L1. We showed high CHI3L1 expression levels in GC tissues and patient sera that significantly correlated with GC progression. Mechanistically, CHI3L1 promoted GC cell growth, proliferation, and metastasis via CHI3L1–CD44 interaction and cascade signaling pathway activation^32^ (Fig. 3).

### Heparin

**Fig. 4:**
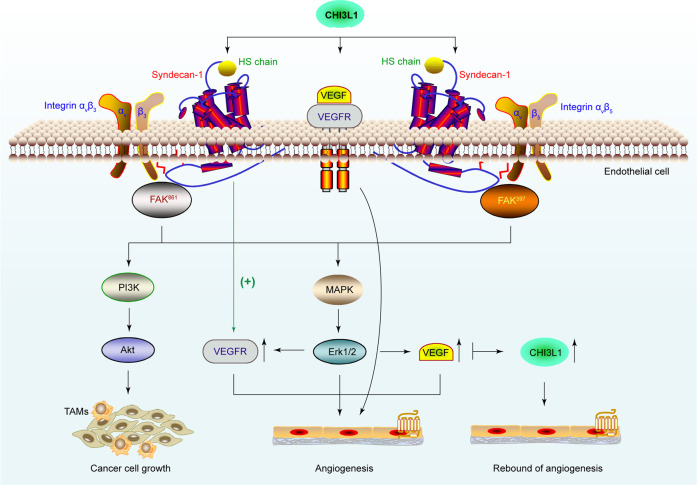
CHI3L1 induces angiogenesis and cancer cell proliferation to facilitate glioblastoma progression. CHI3L1 binds the HS chain of Syn-1 to induce coordination between Syn-1 and the integrin α_v_β_3_, triggering the FAK^861^ and MAPK/Erk1/2 and PI3K signaling pathways to produce the endothelial cell angiogenic signature. Similarly, CHI3L1 induces coordination between Syn-1 and integrin α_v_β_5_ to activate FAK^397^ and downstream signaling pathways, upregulating VEGF. Interestingly, sustained inhibition of VEGF finally upregulates CHI3L1 expression, which contributes to anti-VEGF resistance and invasiveness

CHI3L1 harbors a putative heparin-binding motif consisting of positively charged arginine (R) and lysine (K) (RRDK; residues 144–147).^99^ However, an X-ray crystallographic analysis of CHI3L1 suggested the lack of heparin-binding affinity in the amino acid-rich motif.^43^ A KR-rich domain (residues 334–345) in the C-terminus, rather than the typical RRDK domain, was identified as the functional domain responsible for heparin binding, and the biological activity of CHI3L1.^100^ CHI3L1 interacts with other heparin-like molecules such as heparin sulfate (HS) in the ECM or on the cell surface, which is of paramount importance in cell differentiation, adhesion, proliferation, migration, growth factor and cytokine action, tissue morphogenesis and organogenesis, and tissue injury and remodeling.^99^ Syn-1, an integral membrane protein distributed on the cell surface and in the ECM, is the primary source of cell-surface HS.^101^ Consequently, CHI3L1 induces the coordination between the receptor Syn-1 and the integrin α_v_β_3_, which binds to the HS chain in the ectodomain of Syn-1, triggering FAK^861^ and MAPK/Erk1/2 signaling pathways to enhance the cancer cell growth and produce the endothelial cell angiogenic signature.^34^ CHI3L1 also induces the coordination of Syn-1 and integrin α_v_β_5_, phosphorylating FAK^397^ and Erk1/2 to upregulate VEGF and enhance angiogenesis^102^ (Fig. 4).

### Chitin

Chitin, a glucose derivative, is the primary structural component of cell walls of plants, algae, fungi, and bacteria; the microfilarial sheath of parasitic nematodes; the radulae of molluscs; cephalopod beaks; fish scales; and lissamphibians.^103,104^ CHI3L1 lacks chitinase/hydrolase activity and strongly binds to chitin.^2^ The binding of chitin fragments to CHI3L1 relies on the length of the oligosaccharide. Chitin disaccharides tend to occupy the distal subsites, while longer chitin fragments always occupy the central subsites in the groove.^43^ The absence of enzymatic activity in CHI3L1 is ascribed to a single-residue substitution in the chitinase-3-like catalytic domain, in which an essential glutamic acid is replaced by leucine.^43^ Although chitin has not been identified in mammals to date, it is likely that CHI3L1 interacts with other endogenous substances containing chitin-like motifs.

### Hyaluronic acid

Hyaluronic acid, an anionic and non-sulfated glycosaminoglycan, is widely distributed throughout epithelial, connective, and neural tissues. As one of the chief components of the ECM, hyaluronic acid contributes significantly to inflammation,^105^ wound healing,^106^ granulation tissue formation,^107^ proliferation, and migration.^108^ CHI3L1, along with hyaluronic acid, is increasingly being acknowledged as an effective, non-invasive biomarker for the diagnosis of hepatic fibrosis.^109^ The amino-acid sequence analysis indicated that CHI3L1 contains two potential hyaluronic acid-binding motifs (residues 147–155 and 369–377) on the external face of the folded protein, but this warrants further evaluation using crystallization studies.^66,99^

### Collagen

Affinity chromatography experiments on purified CHI3L1 have demonstrated that it binds all three forms of collagen—types I, II, and III—specifically.^110^ The binding of the chondrocyte-derived species to type I collagen has been demonstrated by surface plasmon resonance analysis and blocking assays.^110^ However, the roles of these interactions require further investigation.

## CHI3L1 in oncogenesis

**Fig. 5:**
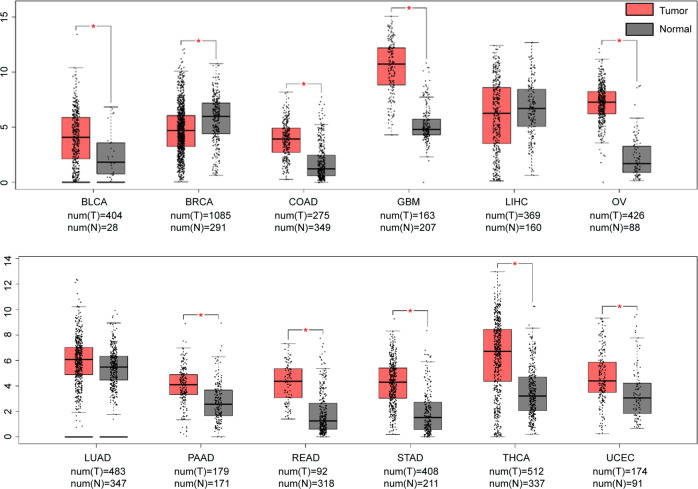
CHI3L1 is overexpressed in certain cancer types. The level of CHI3L1 is significantly overexpressed in tumors including BLCA, COAD, GBM, OV, PAAD, READ, STAD, THCA, and UCEC. However, it is downregulated in BRCA, and shows no difference among LIHC and LUAD (from http://gepia.cancer-pku.cn). *p<0.05. BLCA bladder urothelial carcinoma, BRCA breast invasive carcinoma, COAD colon adenocarcinoma, GBM glioblastoma multiforme, LIHC liver hepatocellular carcinoma, LUAD lung adenocarcinoma, OV ovarian serous cystadenocarcinoma, PAAD pancreatic adenocarcinoma, READ rectum adenocarcinoma, STAD stomach adenocarcinoma, THCA thyroid carcinoma, UCEC uterine corpus endometrial carcinoma

**Fig. 6:**
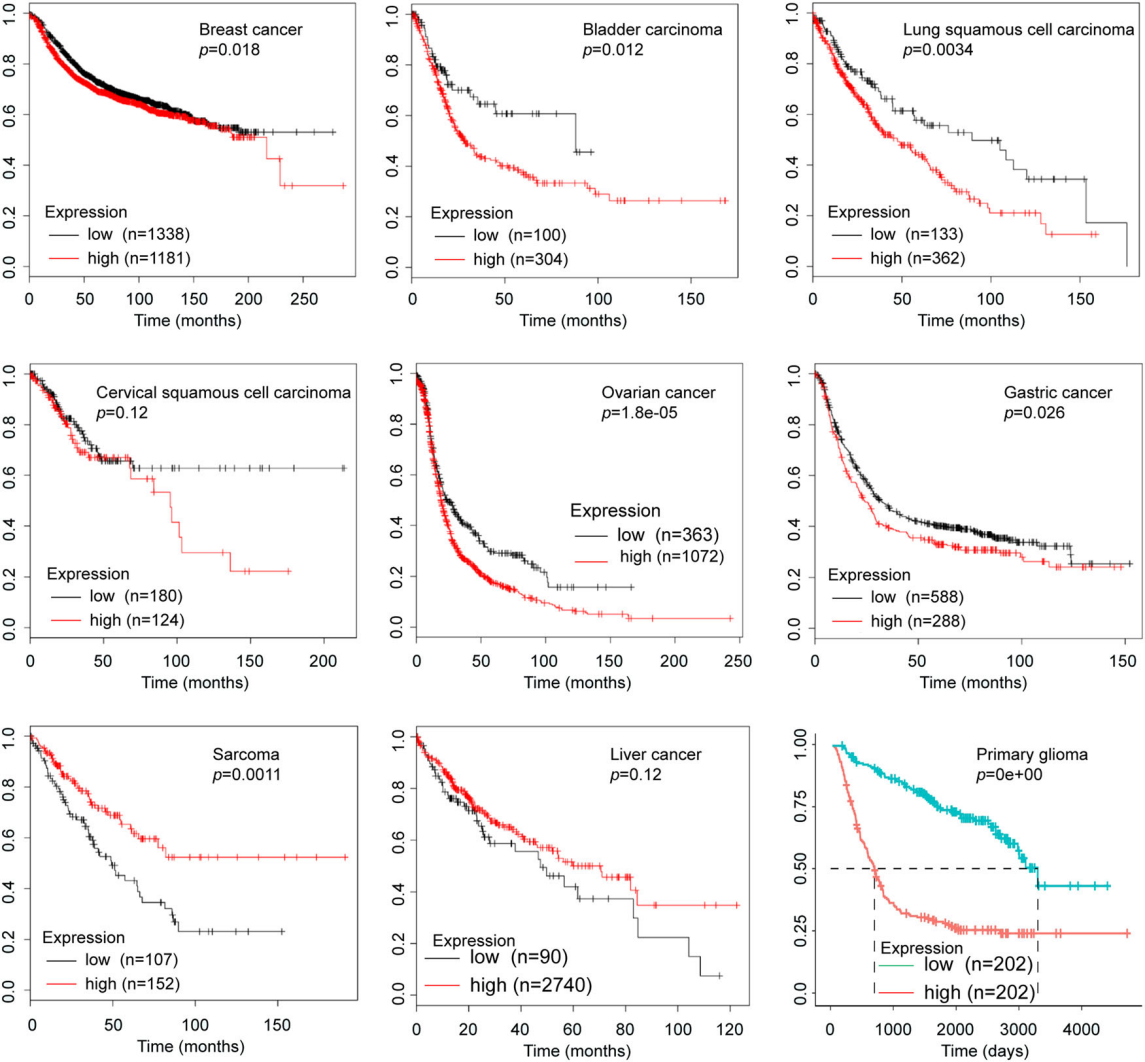
Elevated expression CHI3L1 is inversely correlated with the survival of patients with malignancy. CHI3L1 is overexpressed in patients with breast cancer, bladder carcinoma, lung squamous cell carcinoma, ovarian cancer, gastric cancer, and glioma, and is associated with lower survival rates. Conversely, no relationship is found between CHI3L1 overexpression and survival in patients with cervical squamous cell carcinoma and liver cancer, while a negative correlation was observed between among patients bearing sarcoma. (from https://kmplot.com, http://www.cgga.org.cn)

CHI3L1 is overexpressed in a multitude of human cancers and animal tumor models (Table 2). Moreover, elevated levels of CHI3L1 have been strongly correlated with stages and outcomes of multiple types of primary and secondary carcinomas including oligodendroglia, glioblastoma, osteosarcoma, acute myeloid leukemia, sarcoma, Hodgkin lymphoma, germ-cell, lung, uterine, ovarian, bladder, prostate, kidney, colon, and GCs.^20,22–24,111–116^ As shown in Fig. 5, we analyzed the data from GEPIA (http://gepia.cancer-pku.cn) and found that CHI3L1 level is significantly increased in tumors including bladder urothelial carcinoma (BLCA), colon adenocarcinoma (COAD), glioblastoma multiforme (GBM), ovarian serous cystadenocarcinoma (OV), pancreatic adenocarcinoma (PAAD), rectum adenocarcinoma (READ), stomach adenocarcinoma (STAD), thyroid carcinoma (THCA), and uterine corpus endometrial carcinoma (UCEC). Importantly, increased CHI3L1 level is in significantly associated with poor prognosis and known to affect the survival of patients suffering from a variety of tumors including breast cancer, bladder cancer, lung squamous cell carcinoma, ovarian cancer, GC, sarcoma, and glioma, as analyzed from KMplot (https://kmplot.com) and CGGA (http://www.cgga.org.cn) website (Fig. 6).

**Table 2:** CHI3L1 is overexpressed in certain cancer types

Systems	Tumor types	References
Reproductive system	Breast cancer	^23,28,119,150,237,238,270–272^
	Ovarian cancer	^115,239,240^
	Cervical cancer	^125,139,273^
	Endometrial cancer	^274–276^
	Prostatic cancer	^22,124,277,278^
Digestive system	Gastric cancer	^32,113,150^
	Hepatic carcinoma	^279–281^
	Colon cancer	^21,29,114,118,282,283^
Central nervous system	Glioblastoma	^6,41,111,258,284–287^
	Astrocytoma	^62^
Hematologic system	Acute myeloid leukemia	^20^
	Hodgkin lymphoma	^112^
Motor system	Osteosarcoma	^288^
	Multiple myeloma	^289^
Respiratory system	Lung cancer	^4,24,121,290^
Urinary system	Renal carcinoma	^274^
Others	Melanoma	^27,291–293^

### CHI3L1 promotes cancer cell growth, proliferation, invasion, and metastasis

CHI3L1 stimulation results in the phosphorylation of Erk1/2 in 293 cells, U373 cells, and U87 MG cells, thereby leading to proliferation.^117^ Similarly, a recombinant CHI3L1 was reported to significantly enhance the proliferation of SW480 cells, through the activation of MAPK/Erk signaling pathway.^29^ Furthermore, CHI3L1 significantly promotes the proliferation and migration of colon cancer cells, including SW480 and COLO 205 cells, through the NF-κB signaling pathway.^118^ CHI3L1 also induces the expression of pro-inflammatory and pro-tumorigenic molecules, including CCL2, CXCL2, MMP-2, and MMP-9, which contribute to tumor cell growth and proliferation.^28^ Interestingly, the presence of CHI3L1 in the allergen-induced inflammatory lung attracts myeloid cells, which produce pro-tumorigenic cytokines for tumor growth and metastasis.^119^

Metastasis and invasion are essential hallmarks and leading causes of death in patients with aggressive forms of cancer.^120^ Between 39% and 91% of patients with metastases exhibit elevated serum levels of CHI3L1;^121^ and patients overexpressing CHI3L1 show higher tumor metastasis and poor survival. Consequently, CHI3L1 has been proposed as a prognostic biomarker for neoplastic diseases including papillary THCA, breast cancer, prostate carcinoma, lung cancer, and hepatocellular carcinoma.^22,73,122,123^ Previous studies have shown that CHI3L1 induces the directional migration of endothelial cells.^66^ Consistently, CHI3L1 has been characterized by its ability to promote cancer metastasis and invasion in a variety of tumor types including glioblastoma (U87MG), prostate cancer (C4-2B), and cervical cancer (CaSki and HeLa).^41,124,125^ The epithelial–mesenchymal transition (EMT), a key developmental program which generates cells possessing the properties of stem cells, is often activated during cancer metastasis and invasion.^126^ In the non-small cell lung cancer (NSCLC) cell lines CL1-1, H23, H838, CL1-5, and H2009, CHI3L1 regulates the expression of EMT markers including Twist, Snail, Slug, N-cadherin, vimentin, and E-cadherin,^127^ which indicates that CHI3L1 is a major factor in NSCLC metastasis. Likewise, CHI3L1 expression has been positively correlated with the motility and invasiveness of prostate cancer cells (DU145 and PC3), where it directly affects cancer metastasis by regulating the expression of EMT genes.^128^ Both ECM degradation and reduction in cell-ECM adhesion contribute to malignant transformation and local invasiveness. MMP-2, regarded as an initiator of tumor metastasis, acts by breaking down the ECM, promoting the migration of cancer cells.^129^ CHI3L1 knockdown reduces MMP-2 gene expression and affects the adhesiveness of U87 MG cells to ECM remarkably.^41^

### CHI3L1 favors angiogenesis and vasculogenic mimicry (VM) formation in tumors

Angiogenesis, a crucial part of solid tumor development, supplies oxygen and other essential nutrients in a relatively hypoxic microenvironment found at the center of growing neoplasms, facilitating tumor cell proliferation by enhancing oxygenation.^130^ CHI3L1 has been shown to stimulate the directional migration of umbilical vein endothelial cells (HUVEC), which provided the first significant evidence that CHI3L1 functions in tumor angiogenesis by stimulating the reorganization and migration of vascular endothelial cells.^66^ Currently, CHI3L1 is increasingly acknowledged as an angiogenic stimulator, which promotes tumor angiogenesis through VEGF-dependent and VEGF-independent pathways.^102,131,132^

VEGF, a vital signaling protein involved in both vasculogenesis and angiogenesis, has been shown to be one of the most potent mediators of angiogenesis in tumor initiation and promotion.^133^ Serum levels of CHI3L1 correlate with VEGF expression and microvessel density in tumor tissues in a VEGF-dependent manner, which promotes angiogenesis and is responsible for cancer progression.^125^ CHI3L1 also influences the coordination between Syn-1 and integrin α_v_β_5_, thereby upregulating VEGF and enhancing angiogenesis through the phosphorylation of FAK^397^ and Erk1/2.^102^ CHI3L1-induced angiogenesis results from the coordination between integrin α_v_β_3_ and Syn-1, which triggers the FAK^861^ and MAPK/Erk1/2 signaling pathways in a VEGF-independent manner in MDA-MB-231 breast cancer and HCT-116 colon cancer cells.^34^ Alternatively, CHI3L1 enhances the production of pro-angiogenic molecules like CCL2, CXCL2, and MMP-9 in the pulmonary infiltrating macrophages of mammary tumor-bearing mice.^134^

VM is an alternative microvascular system that exhibits high aggressiveness and genetically dysregulates tumor cell orchestration.^135^ It facilitates tumor perfusion and promotes distant metastasis, in the absence of angiogenesis, in highly malignant cancers including melanoma,^136^ small cell lung cancer,^137^ and glioblastoma.^138^ Recently, the expression of CHI3L1 in cervical cancer tissues was shown to be positively associated with VM formation, with CHI3L1 directly stimulating the formation of vessel-like structures in vitro in cervical cancer cells.^139^ However, the mechanism underlying the induction of VM formation by CHI3L1 requires further investigation.

### CHI3L1 drives tumor-associated inflammation

Inflammation is increasingly regarded as one of the hallmarks of cancer.^140^ Inflammatory responses at metastatic sites supply abundant bioactive molecules to the tumor microenvironment including growth factors, survival factors, pro-angiogenic factors, and ECM-modifying enzymes that facilitate invasion and metastasis, and inductive signals that switch on the activation of EMT.^141–143^ Moreover, infiltrating inflammatory cells like TAMs, T cells, and myeloid-derived cells promote angiogenesis and accelerate EMT around the tumor lesion.^144^

CHI3L1 knockout tumor bearers with pre-existing allergen-induced pulmonary inflammation show decreased pro-inflammatory mediator secretion, significantly decreased populations of myeloid-derived cells, reduction in tumor volume, reduced lung metastases, and increased survival relative to wild-type controls.^119^ An investigation exploring the role of CHI3L1 in “pre-metastatic” lungs revealed that CHI3L1 induced the expression of pro-angiogenic molecules including CCL2, CXCL2, and MMP-9 in macrophages to facilitate the incidence of pulmonary metastasis.^134^ In vitro, CHI3L1 efficiently enhanced the secretion of IL-8 and TNF-α from SW480 cells by activating the NF-κB signaling pathway, thereby promoting cancer cell proliferation and migration.^118,145^ Additionally, CHI3L1 stimulation increased the expression of IL-8 and CCL2 by SW480 cells via the activation of the MAPK cascade pathway.^29^ Mechanistically, residues 325–339 of the carbohydrate-binding motif of CHI3L1 specifically bind to IL-8 promoters and enhance IL-8 production in SW480 cells via the phosphorylation of Akt.^145^ IL-8, MMP-2, MMP-9, CCL2, and CXCL2, along with other proinflammatory mediators at the tumor site, favor tumor progression by promoting angiogenesis, accelerating ECM degradation, providing sustained survival signals to cancer stem cells, and recruiting immunosuppressive myeloid cells.^146,147^

The expression of CHI3L1 has been significantly associated with the migration of THP-1 cells and the infiltration of TAMs in colon cancer.^29^ Mechanistically, CHI3L1 promotes macrophage recruitment through the MAPK (particularly Erk and JNK) signaling pathway in cancer cells to secrete inflammatory chemokines, primarily IL-8 and CCL2.^29^ Specialized subpopulations of macrophages, key components in the tumor microenvironment, facilitate cancer cell growth, proliferation, invasion, and metastasis, while inhibiting antitumor immunity.^143^ Upon recruitment and activation, CHI3L1 induces M2 macrophage (alternatively activated) differentiation by the phosphorylation of Erk.^19,148^ Conversely, CHI3L1 is highly expressed in *S. aureus*-stimulated macrophages, where it promotes M1 polarization of macrophages (classically activated) via activation of the MAPK pathway.^149^ Mechanistically, CHI3L1 secreted from M2 macrophages interacts with IL-13Rα2 on the plasma membrane of cancer cells, triggering the activation of the MAPK signaling pathway in GC and breast cancer.^150^ Upon activation, Erk and JNK signaling enhances the expression of MMPs, which degrade the ECM in the tumor microenvironment, thereby facilitating metastasis.^151^ Accordingly, CHI3L1 induces MMP-9 production by macrophages and enhances matrix degradation in triple-negative breast cancer mouse models.^152^ Moreover, the deletion or targeted inhibition of CHI3L1 enhances Th1 effector cytokine (IFN-γ, TNFα) production and CTL response to enhance anti-tumor immunity and reduce pulmonary metastasis.^73^

## CHI3L1 in non-cancerous diseases

CHI3L1 also played critical roles in the pathogenesis of non-cancerous diseases. A recent study followed prospectively 94,665 individuals from the Danish general population for up to 23 years and analyzed for plasma CHI3L1 levels (*n* = 21,584) and CHI3L1 rs4950928 genotype (*n* = 94,184), and reveals that baseline elevated plasma CHI3L1 is not a cause, but a strong marker of increased risk of future infectious diseases in individuals in the general population.^153^ The roles of CHI3L1 are further summarized according to the organ systems of the human body in Table 3.

**Table 3:** CHI3L1 in other non-cancerous diseases

System	Category	Related factors	Phenomenon and effect
Respiratory	Inflammation	Cigarette smoke^33^	CHI3L1↑
		RSV^154^	CHI3L1↑ → M2 MΦ activation
		IL-18^155^	CHI3L1↑ → Type 2 and type 17 inflammation and fibrotic airway remodeling
		Interstitial lung disease^157,158^	CHI3L1↑ → poorer prognosis
		Chronic inflammatory airway disease^294^	CHI3L1↑ → MUC5AC↑
		Bacterial infection^16^	CHI3L1↑ → bacterial clearance
		Hypersensitivity pneumonitis^156^	CHI3L1↑ → poorer prognosis
	Ashma	High fat diet^74^	CHI3L1↑ → WAT accumulation and lung Th2 inflammation
		Bronchial remodeling^33^	CHI3L1↑ → bronchial smooth muscle cell proliferation and migration
		Asthma status and lung function^159–162^	CHI3L1↑ → asthma↑, lung function↓
	Lung fibrosis	Idiopathic pulmonary fibrosis^163^	CHI3L1↑ → injury↓ profibrotic effect ↑
		Hermansky-Pudlak syndrome^31,97^	CHI3L1↑ → pulmonary fibrosis↑
		Cystic fibrosis^164^	CHI3L1↑ → exacerbations↑
		Asbestosis^165^	CHI3L1↑ → lung function↓
	Lung injury	Hyperoxia^30,269^	CHI3L1↓ → acute lung injury↑
		Oxidant-induced^30^	CHI3L1↑ → oxidant-induced apoptosis and lung injury
	COPD	Status and airway remodeling^166,167^	CHI3L1↑ → exacerbations↑, MΦ activation↑
Digestive	Liver injury	ConA^168^	CHI3L1 → liver injury↑
		APAP^169^	CHI3L1 → liver damage↓
		Alcohol^170^	CHI3L1 → liver injury↑
		Ischemia–reperfusion^171^	CHI3L1 → liver injury↑
	Liver fibrosis	Hepatitis C	CHI3L1↑ → fibrosis rate↑, TGF-β↑;^172^ Promoter polymorphism is not associated with disease progression;^175^ CHI3L1↑ → false-positive rates;^176^ CHI3L1↑ → Rapid fibrosis progression after liver transplantation;^173^ CHI3L1↑ → steatosis↑^174^
		Hepatitis B^177–180^	Serum CHI3L1↑ → liver fibrosis
		Alcoholic liver disease^181,182^	CHI3L1↑ → liver fibrosis
		Non-alcoholic fatty liver disease^183^	CHI3L1↑ → liver fibrosis
	Bowel diseases	IBD	Fecal CHI3L1 → mucosal inflammation^184^ and endoscopic activity^185^
		Colitis	CHI3L1 binds to bacterial chitin-binding protein,^186,187^ enhances bacterial adhesion and invasion,^60^ activates Akt signaling^145^ and IL-6-mediated STAT3 Phosphorylation^188^
Cardiovascular	Atherosclerosis		CHI3L1↑ → coronary^189,190^ and carotid^191^ atherosclerosis severity↑, risk↑,^193^ exacerbates atherosclerosis^192^
	Coronary artery disease	Type 1^194^ and type 2^195^ diabetes	CHI3L1↑
	Peripheral artery disease		CHI3L1↑ → risk↑,^196,197^ ankle-brachial index↓^198^
	Giant cell arteritis		CHI3L1↑ in giant cells and macrophages^199^
	Thromboembolism	Venous^201^ and incident^200^	CHI3L1↑
	Hypertension	Chinese men^295^	CHI3L1↑
		idiopathic pulmonary arterial Hypertension^202^	CHI3L1↑
		Obstructive sleep apnea (OSA)	CHI3L1↑ → endothelial function↓
	Atrial fibrillation		CHI3L1↑ in epicardial adipose tissue,^205^ risk↑^204^
	β-thalassemia major	Hepatic fibrosis	CHI3L1↑^206^
	Ischemic heart disease	Type 2 diabetes	CHI3L1↑^195^
	Chronic heart failure		CHI3L1↑^207^
Endocrine	Diabetes	Type 2	CHI3L1↑^209^ → BMI-independent marker,^213^ albuminuria,^211,212^ mortality,^214^ obesity,^210^ psychotic disorders^296^
		Type 1	CHI3L1↑ → albuminuria^215^
	Obesity		CHI3L1↑^208^
	Insulin resistance		CHI3L1↑ → TNFα-induced inflammation and insulin resistance↓^9^
Nervous	AD		CSF CHI3L1↑,^216,217,219–222^ No change^223,224^
	PD		CHI3L1↑ → cognitive function↓^226^
Urinary	Renal disease	Kidney injury	CHI3L1↑in pediatric severe malaria,^229^ ischemic injury,^17^ cardiac surgery-associated,^230^ adult critically ill,^228^ hospitalized patients^227^
		Fibrosis^232^	CHI3L1↑
		Hemodialysis^234^	CHI3L1↑ → mortality risk↑
		Nephrotic syndrome^235^	CHI3L1↑ → endothelial dysfunction and increased arterial stiffness
	Bladder	Bladder pain syndrome, interstitial cystitis s^233^	CHI3L1↑
Skeletal	Joint	Arthritis	CHI3L1↑^236^
		Osteoarthritis	CHI3L1↑^47,297–300^
		Rheumatoid arthritis	CHI3L1↑,^241–246,301^ autoantigen,^248–250^ autoantibody,^252^ targeted therapy^247,251,259,260^

## Respiratory diseases

### Inflammation

Elevation of CHI3L1 level is induced by inflammations in lungs. The causes behind inflammation include cigarette smoke,^33^ virus,^154^ and bacterial^16^ infection. CHI3L1 regulates M2 macrophage activation and Th2 immune response during RSV infection.^154^ CHI3L1 also promotes *Streptococcus pneumoniae* bacteria clearance by inhibiting caspase-1-dependent macrophage pyroptosis, and augments host tolerance to lung antibacterial responses by controlling inflammasome activation, ATP accumulation, and production of thymic stromal lymphopoietin and type 1, type 2, and type 17 cytokines.^16^ In the absence of CHI3L1, IL-18-induced Type 2 and Type 17 inflammation and fibrotic airway remodeling were significantly ameliorated while Type 1 inflammation and emphysematous alveolar destruction were enhanced.^155^ Instead, CHI3L1 increases the expression of MUC5AC, which is the major mucin in the human respiratory tract leading to chronic cough and sputum production in chronic inflammatory airway disease. Moreover, high levels of serum CHI3L1 predicts disease progression and are associated with mortality of hypersensitivity pneumonitis.^156^ Furthermore, CHI3L1 has been regarded as a promising biomarker for evaluating severity of interstitial lung disease and predicting disease prognosis.^157,158^

### Asthma

CHI3L1 has been found in increased quantities in the serum and lungs in patients with asthma.^159–161^ Moreover, a promoter SNP (−131C → G) in *CHI3L1* is associated with elevated serum CHI3L1 levels, asthma, bronchial hyper responsiveness, and measures of pulmonary function.^162^ Interestingly, CHI3L1 is induced by a high fat diet and contributes to the genesis of obesity and asthma.^74^ Mechanistically, CHI3L1 promotes bronchial smooth muscle cell proliferation and migration through a PAR-2–dependent mechanism.^33^

### Lung injury and fibrosis

CHI3L1 has been reported to ameliorate hyperoxic acute lung injury and prolong mouse survival in 100% O_2_.^30,68^ In mammalian lung fibrosis CHI3L1 plays a profibrotic role in the repair phase by augmenting alternative macrophage activation, fibroblast proliferation, and matrix deposition.^163^ Additionally, CHI3L1 exacerbates HPS-associated pulmonary fibrosis through binding to CRTH2 receptor.^31,97^ Serum CHI3L1 levels are also increased in patients with cystic fibrosis^164^ as well as asbestosis.^165^

### Chronic obstructive pulmonary disease (COPD)

CHI3L1 is upregulated in COPD,^166,167^ in which it may contribute to tissue inflammation and remodeling by sustaining the synthesis of proinflammatory and fibrogenic chemokines and of metalloproteinases by alveolar macrophages.^166^

## CHI3L1 in digestive diseases

### Liver injury

There are different types of liver injury. In concanavalin A-induced liver damage, CHI3L1 promotes intrahepatic activation of coagulation and tissue injury through induction of tissue factor via MAPK activation.^168^ In acetaminophen-induced liver damage, CHI3L1 deficiency results in more severe liver injury.^169^ Moreover, the lack of CHI3L1 attenuates ethanol-induced liver injury by inhibition of sterol regulatory element-binding protein 1-dependent triglyceride synthesis.^170^ Additionally, ischemia-reperfusion injury in steatotic livers following transplantation are associated with MMP activation and CHI3L1 upregulation resulting in pro-fibrotic and proinflammatory cytokine release.^171^

### Liver fibrosis

Chronic infection with hepatitis virus predisposes to liver fibrosis and end-stage liver complications. For HCV infection, it has been reported that the progression of fibrosis rate/year has a direct linear correlation for CHI3L1 which also shows a linear correlation with TGF-β in patients with concomitant HCV and schistosomiasis infection.^172^ Moreover, elevated levels of serum CHI3L1 within the first 6 months after liver transplantation accurately predict rapid fibrosis progression.^173^ CHI3L1 remains associated with steatosis after controlling for fibrosis in Egyptian patients with HCV infection.^174^ Additionally, a functional upstream promoter polymorphism of CHI3L1 (rs4950928) in a large cohort of German patients with chronic HCV is associated with a lower stage of liver fibrosis as well as lower serum CHI3L1 levels.^38^ However, this promoter polymorphism is not associated with disease progression in patients in the United States with advanced fibrosis due to chronic HCV.^175^ Although serum CHI3L1 level is correlated with the Ishak stages of fibrosis and predicts advanced fibrosis and cirrhosis, it shows the false-positive rates in discriminating three clinically relevant stages of fibrosis.^176^ For hepatitis B virus (HBV) infection, serum ChI3L1 level is a feasible biomarker to identify advanced liver fibrosis in patients with HBV-related liver fibrosis.^177–179^ Moreover, CHI3L1 is regarded as a potential useful marker for monitoring the change of liver fibrosis in patients with chronic HBV infection during therapy.^180^ For alcohol-induced fibrosis, increased serum CHI3L1 in patients with liver disease of various degree and etiology seems to reflect fibrosis and fibrogenesis.^181,182^ Furthermore, in non-alcoholic fatty liver disease macrophage-derived CHI3L1 is also judged as a feasible biomarker of liver fibrosis in patients.^183^

### Bowel disease

For inflammatory bowel disease (IBD), fecal CHI3L1 has been reported as a novel biomarker of disease activity in pediatric patients^184^ and endoscopic activity in adult patients.^185^ For colitis CHI3L1 exacerbates intestinal inflammation by binding to bacterial chitin-binding protein,^186,187^ enhancing bacterial adhesion and invasion,^60^ activating Akt signaling^145^ and IL-6-mediated STAT3 phosphorylation.^188^

## CHI3L1 in cardiovascular diseases

### Atherosclerosis, coronary artery disease, peripheral artery disease, and giant cell arteritis

Plasma CHI3L1 levels correlate with the severity of coronary^189,190^ and carotid^191^ atherosclerosis. Moreover, atherosclerosis is exacerbated by CHI3L1 in amyloid precursor protein transgenic mice.^192^ Accordingly, *CHI3L1* gene silencing could downregulate the expression of local proinflammatory mediators and inhibit plaques progression.^193^ Additionally, CHI3L1 is an early inflammatory marker in diabetic subjects even in the presence of a low atherosclerotic background,^194,195^ and is elevated in patients with peripheral arterial disease and diabetes or pre-diabetes.^196,197^ It has been revealed that CHI3L1 levels increase with declining ankle-brachial index and are associated with long-term cardiovascular mortality in peripheral arterial disease patients.^198^ For giant cell arteritis, CHI3L1 is found in CD68^+^ giant cells and mononuclear cells in the media of arteritic vessels of patients, and serum CHI3L1 level reflects the local activity of these cells in the inflamed artery.^199^

### Thromboembolism, hypertension, atrial fibrillation, β-thalassemia major, and chronic heart failure

It has been reported that baseline plasma CHI3L1 level is significantly associated with incident thromboembolic stroke with a magnitude of effect.^200^ Similarly, high CHI3L1 levels are associated with a 2-fold increased risk of venous thromboembolism.^201^ Additionally, CHI3L1 is associated with hypertension incidence only among men in China. Moreover, in pulmonary arterial hypertension plasma CHI3L1 levels are significantly increased and regarded as a prognostic indicator.^202^ Furthermore, CHI3L1 has a potential for being a biomarker for endothelial dysfunction and hypertension in obstructive sleep apnea.^203^ For atrial fibrillation, elevated plasma CHI3L1 levels are robustly associated with its increased risk originating from hospital admissions or visits to the emergency department.^204^ Interestingly, CHI3L1 is highly expressed in the epicardial adipose tissue of patients with atrial fibrillation and associated with atrial fibrosis.^205^ Even in β-thalassemia major patients CHI3L1 is shown as a promising marker of cardiovascular disease and liver siderosis.^206^ In patients with chronic heart failure, high level of serum CHI3L1 is associated with higher rates of cardiac events and regarded as an independent prognostic factor.^207^

## CHI3L1 in endocrine diseases

### Obesity, insulin resistance and diabetes

Low-grade chronic inflammation is associated with obesity and type 2 diabetes. CHI3L1 functions as an inflammatory regulator with relation to acute and chronic inflammation, and has played an important role in diabetes. CHI3L1 levels are elevated in morbidly obese patients^208^ and patients with type 2 diabetes,^209,210^ is related to insulin resistance,^209^ is independently associated with albuminuria,^211,212^ and is a BMI-independent marker.^213^ Moreover, high CHI3L1 levels predict mortality in patients with type 2 diabetes.^214^ CHI3L1 is also elevated in patients with type 1 diabetes and increases with levels of albuminuria.^215^

## CHI3L1 in nervous diseases

### Alzheimer’s disease (AD) and Parkinson’s disease (PD)

CHI3L1 is firstly identified as a potential candidate cerebrospinal fluid (CSF) biomarker for AD by using two-dimensional difference gel electrophoresis and liquid chromatography tandem mass spectrometry.^216^ The diagnostic function of CHI3L1 in AD and other neurodegenerative diseases has been comprehensively summarized by Harald Hampel and his colleagues.^217^ Plasma CHI3L1 levels are not elevated in moderate/severe AD, suggesting that plasma CHI3L1 increase probably occurred in early AD phases.^218^ There is growing evidence suggesting that CSF CHI3L1 might be of diagnostic value in distinguishing AD from healthy controls. However, some studies reported higher CSF CHI3L1 concentrations in AD versus controls,^219–222^ while no significant differences were reported in other independent analyses.^223,224^ A meta-analysis compared CSF CHI3L1 in six different cohorts of AD patients and five cohorts of normal controls demonstrating a moderate significant effect size.^217,225^ For PD, a significant increase of CSF CHI3L1 concentrations was observed in PD patients, after a 2-year follow-up, compared with baseline but not in healthy controls, and was associated to a faster cognitive decline in PD versus healthy controls.^226^

## CHI3L1 in urinary diseases

CHI3L1 has been identified as a critical mediator that limits tubular cell apoptotic death and improves animal survival after kidney ischemia/reperfusion by urine proteomic screen, thereby serving as a sensor of the degree of injury and a possible biomarker to identify patients at greatest risk of sustained renal failure after transplantation.^17^ Urine CHI3L1 is also associated with acute kidney injury (AKI) progression and/or death in hospitalized patients and improves clinically determined risk reclassification.^227^ Moreover, urine CHI3L1 is regarded as a biomarker for prediction of AKI stage ≥2 in adult ICU patients.^228^ Especially, CHI3L1 is thought as a novel biomarker of malaria-associated AKI and an independent risk factor for mortality that is associated with well-established pathways of severe malaria pathogenesis including inflammation, endothelial activation, and hemolysis.^229^ A single-center prospective cohort study indicates that serum CHI3L1 combined with urine CHI3L1 is a good predictor of AKI associated with elective cardiac surgery at stage ≥2 within 12 h after the time of post-operative ICU admission.^230^ In chronic kidney disease, urinary CHI3L1 is associated with higher risk of the kidney composite outcome in fully adjusted models including baseline eGFR and urine albumin.^231^ CHI3L1 has been reported to promote renal fibrosis after kidney injury via activation of myofibroblasts.^232^ In bladder pain syndrome/interstitial cystitis serum and urine levels of CHI3L1 are suggested as non-invasive biomarkers for the evaluation of bladder fibrogenesis.^233^ In hemodialysis patients CHI3L1 significantly improves risk prediction for all-cause and cardiovascular mortality.^234^ In nephrotic syndrome patients the serum CHI3L1 level is associated with endothelial dysfunction and increased arterial stiffness and may be an indicator of the level of proteinuria in this patient population.^235^

## CHI3L1 in skeletal diseases

### OA and rheumatoid arthritis

CHI3L1 is expressed in diseased human osteoarthritic cartilage and osteophyte,^39^ and is found to induce arthritis accompanied by pathologic changes in bone and cartilage.^236^ There are many studies indicating CHI3L1 as a cartilage-derived factor associated with mediators of inflammation and cartilage destruction involved in the pathogenesis of OA.^237–240^ For rheumatoid arthritis (RA), serum CHI3L1 in the RA patient group is significantly higher than that in the other patient groups and healthy controls,^241,242^ is increased in 54% of the patients with clinically active disease,^243^ and elevated serum CHI3L1 is related to progression in joint destruction in early RA patients.^244,245^ CHI3L1 in synovial fluid influences serum CHI3L1. Both are involved in the pathophysiology of the arthritic processes and reflect local disease activity.^246^ Moreover, CHI3L1 has been identified as a candidate autoantigen presented by HLA-DR in RA.^247–250^ Some patients with RA and OA possessed autoantibodies to CHI3L1.^251,252^ However, autoimmunity to CHI3L1 in patients with OA was present at equal or somewhat higher frequency than in patients with RA. The cellular and humoral immune responses to CHI3L1 may be involved in the pathological process of OA as well as RA.^252^

## Targeting CHI3L1 for therapy

### Tumor

Owing to its overexpression in a wide array of cancer types, CHI3L1 is now being regarded as a potential diagnostic marker and therapeutic target in oncology.^253,254^ For example, CHI3L1 is overexpressed in glioblastoma patients with poor survival. Encouragingly, a CHI3L1-neutralizing antibody effectively inhibits tube formation of microvascular endothelial cells, abolishes the CHI3L1-induced VEGF receptor 2 expression, and accelerates the apoptosis of glioblastoma U87 cells induced by γ-irradiation exposure through the blockade of Akt pathway.^255^ Glioblastoma U87 cells produce increased amounts of CHI3L1 during γ-irradiation-induced cell death, however, the blockade of CHI3L1 activity using a monoclonal neutralizing antibody decreased tumor growth, angiogenesis, and metastasis in a xenograft model.^102^ Consequently, a combination of CHI3L1-neutralizing antibody and ionizing irradiation synergistically inhibited tumor growth and increased mouse survival relative to single treatment in xenografted brain tumor mouse models.^131^ In temozolomide-resistant (TMZ-R) glioblastoma, CHI3L1 inhibition suppressed invasive activity and partially restored the sensitivity to TMZ.^63^ The blockade of STAT3 by STX-0119 resulted in decreased CHI3L1 expression and inhibition of TMZ-R U87 cell growth.^256^ Targeting the CHI3L1-STAT3-mTOR signaling pathway using a combination of the mTOR inhibitor rapamycin and a STAT3 inhibitor produced a significant growth-inhibitory effect in TMZ-R relapsed gliomas.^257^ In colon cancer, the migration of SW480 cells was significantly enhanced in the presence of CHI3L1, but was markedly inhibited by anti-CHI3L1 antibody treatment.^118^ Similarly, the addition of anti-CHI3L1 antibody in culture resulted in a significant decrease in the adhesion, migration, and invasion of GC (MKN-45) and breast cancer (MDA-MB-231) cells.^150^ In particular, a neutralizing anti-CHI3L1 antibody targeting the KR-rich domain (residues 334–345) abrogated angiogenesis and tumor cell migration in breast cancer.^100^

In addition to anti-CHI3L1-specific antibody and targeted chemical inhibitors, pan-family 18 chitinase inhibitors like chitin display identical blocking effect induced by CHI3L1. In mammary tumor-bearing mouse model, chitin treatment reduced the induction of proinflammatory mediators (CCL2, CXCL2, and MMP-9), tumor growth, and pulmonary metastasis induced by CHI3L1 overexpression.^28^ Moreover, resveratrol, a natural phenol, decreased the activity of the CHI3L1 promoter, reducing both mRNA transcription and protein expression, thereby repressing the growth, proliferation, and invasion of U87 MG cells in vitro.^258^

### Non-cancerous diseases

It is now well-established that CHI3L1 plays a crucial role in the pathogenesis of many types of non-cancerous diseases. Therefore, CHI3L1 could serve as a potential therapeutic target. For instance, CHI3L1 is required for severe lung immunopathology caused by RSV infection. Consequently, in vivo neutralization of CHI3L1 using an anti-CHI3L1 antibody decreased the severity of IL-13-dominant airway inflammation during RSV infection.^154^ In IBD CHI3L1 enhances bacterial adhesion and invasion on/into CECs. Accordingly, inhibition of CHI3L1 by anti-CHI3L1 antibody or CHI3L1-specific short interfering RNA reduces the adhesion of chitin-binding protein overexpressing *E. coli* to CECs.^226^ In RA CHI3L1 is identified as a HLA-DR-restricted autoantigen. The antibodies against CHI3L1 (263–275) peptide are able to inhibit (up to 90%) the response of the peptide-specific HLA-DR-restricted T cell hybridomas to peptide-pulsed APC or purified complexes.^259^ Moreover, inhalation of CHI3L1 protein leads to tolerization of antigen-specific T cells and to suppression of CHI3L1-induced arthritis in mice.^247^ Subsequently, the safety and tolerability and pilot efficacy of repeated single doses of Org39141 (recombinant human CHI3L1) by intranasal administration was performed by a phase I escalating cohort study in patients with RA.^260^ As a consequence, Org 39141 is well tolerated, and no severe or serious adverse events is reported. After 4 weeks of treatment, the mean decrease in Org 39141 treatment group (−24%) is statistically (*p* = 0.02) and clinically significantly larger than in the pooled placebo group (−3%).^260^

In summary, considering the multiple roles of CHI3L1 in oncogenesis, the direct neutralization of CHI3L1 will reduce tumor-associated inflammatory response, ECM degradation, angiogenesis, and tumor metastasis, thereby inhibiting tumor progression.^150,261^ Moreover, considering the binding partners involved in CHI3L1 signaling, reagents targeting and disrupting these interactions warrant further investigation in future cancer research and clinical trials. Likewise, considering the effects of CHI3L1 in pathogenesis of non-cancerous diseases, the techniques blocking the function of CHI3L1 including neutralizing antibodies, small interfering RNA and microRNA can be introduced to ameliorate the disease symptoms. Since CHI3L1 has been revealed as an autoantigen in RA, the recombinant protein can be employed to induce mucosal tolerance by intranasal administration for further clinical development.

## Closing thoughts

Much has been known about the effect of CHI3L1 in disease pathologies in the past 30 years. It is perhaps tempting to ponder how we might integrate this knowledge into clinical applications. However, some puzzles still need to be solved.

First, what are all functional domains or structures in CHI3L1 molecule? CHI3L1 has a triose-phosphate isomerase barrel-like structure with the insertion of beta-strands domain, and binds chitin and chito-oligosaccharides using nine GlcNAc-binding subsites. A chemical candidate was identified as a CHI3L1 inhibitor to attenuate NF-κB activation and NF-κB-related neuroinflammatory gene expression,^65^ and the binding sites were further analyzed by a docking model.^262^ It was found that the antibody against CHI3L1 (325–339) peptide reduced the adhesion of chitin-binding protein-overexpressing *E. coli* to CECs,^186^ thereby revealing the importance of the region (325–339) in the CHI3L1 structure. Once the functional domains or structures are fully identified, the chemicals or antibodies will be able to be designed or produced to target them precisely.

Second, whether is the function of CHI3L1 redundant? CHI3L2 is a protein of unknown function closely related to CHI3L1 sharing 51% identities and 71% positives and present in humans and other primates. CHI3L3 (Ym1) and CHI3L4 (Ym2) are proteins with unknown function closely related to CHI3L1 in mice, sharing 43% identities and 61% positives, 42% identities and 59% positives to CHI3L1, respectively. Once the functional redundancy of all proteins is determined, the targets may be expanded accordingly to eliminate all effects.

Third, are there any as-yet unknown receptors or ligands of CHI3L1? Although a few receptors have been identified, there still might be novel ones. Especially, some membrane proteins with functional intracellular domains may function as CHI3L1 receptors. Probably, the protein complexes need to be formed and function in CHI3L1 signaling.

Forth, how does CHI3L1 signaling influence neutrophils, monocytes, or macrophages recruitment? CHI3L1 participates in the infiltration of neutrophils in lung^69^ and in liver (our unpublished data). Although some kinases have been reported to be activated and involved, the direct functional receptors remain to be further investigated.

**Fig. 7:**
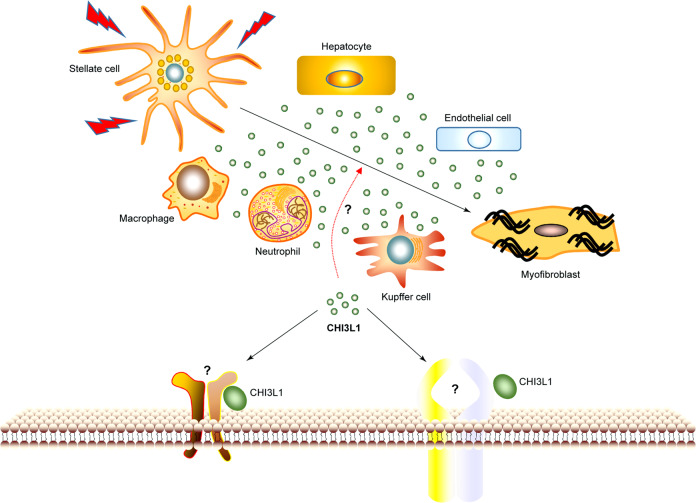
Possible effects of CHI3L1 on liver fibrosis

Fifth, what is the exact function of CHI3L1 in liver fibrosis? The cellular sources of CHI3L1 in liver include hepatocytes, neutrophils, macrophages, and endothelial cells (Fig. 7). Numerous studies show that CHI3L1 upregulates in liver fibrosis caused by virus infection, alcohol, or the accumulation of liver lipid. A recent paper shows that CHI3L1 deficiency ameliorates liver fibrosis by promoting hepatic macrophage apoptosis.^262^ However, the exact effect or the involved receptors still remain to be determined. The success of CHI3L1-targeted therapies will depend on whether we can differentiate CHI3L1 functions and its receptors in various biological and pathological responses.
